# Parenting experiences of couples living with human immunodeficiency virus: A qualitative study from rural Southern Malawi

**DOI:** 10.1080/17290376.2014.886140

**Published:** 2014-05-12

**Authors:** Belinda Chimphamba Gombachika, Johanne Sundby, Ellen Chirwa, Address Malata

**Affiliations:** ^a^ PhD Nursing, is a PhD candidate at the Department of Community Medicine, Institute of Health and Society, University of Oslo, Oslo, Norway; ^b^ PhD Community Medicine, Professor at the Department of Community Medicine, Institute of Health and Society, University of Oslo, Oslo, Norway; ^c^ PhD Nursing Science, Vice Principal at the Faculty of Nursing, Kamuzu College of Nursing, University of Malawi, Blantyre Campus, Blantyre, Malawi; ^d^ PhD Nursing, Principal at the Faculty of Nursing, Kamuzu College of Nursing, University of Malawi, Lilongwe Campus, Lilongwe, Malawi

**Keywords:** Malawi, couples, experiences, HIV and AIDS, parenting, Malawi, les couples, les expériences, le VIH et le SIDA, la parentalité

## Abstract

The advent of antiretroviral therapy (ART) has allowed couples living with human immunodeficiency virus (HIV) to live longer and healthier lives. The reduction in the mother-to-child transmission of HIV has encouraged some people living with HIV (PLWH) to have children. However, little is known about the parenting experiences of couples living with HIV (CLWH). The aim of this qualitative study was to explore and describe parenting experiences of seroconcordant couples who have a child while living with HIV in Malawi. Data were collected using in-depth interviews with 14 couples purposively sampled in matrilineal Chiradzulu and patrilineal Chikhwawa communities from July to December 2010. The research findings shows that irrespective of kinship organization, economic hardships, food insecurity, gender-specific role expectations and conflicting information from health institutions and media about sources of support underpin their parenting roles. In addition, male spouses are directly involved in household activities, childcare and child feeding decisions, challenging the existing stereotyped gender norms. In the absence of widow inheritance, widows from patrilineal communities are not receiving the expected support from the deceased husband relatives. Finally, the study has shown that CLWH are able to find solutions for the challenges they encounter. Contrary to existing belief that such who have children depend solely on public aid. Such claims without proper knowledge of local social cultural contexts, may contribute to stigmatizing CLWH who continue to have children. The study is also relevant to PLWH who, although not parents themselves, are confronted with a situation where they have to accept responsibility for raising children from their kin. We suggest the longer-term vision for ART wide access in Malawi to be broadened beyond provision of ART to incorporate social and economic interventions that support the rebuilding of CLWH social and economic lives. The interventions must be designed using a holistic multi-sector approach.

## Résumé

L'avènement de la thérapie antirétrovirale a permis couples vivant avec le VIH de vivre plus longtemps et en meilleure santé. La réduction de la transmission du VIH de la mère à l'enfant a encouragé certaines personnes vivant avec le VIH ont deux enfants. Cependant, on sait peu sur les expériences parentales des couples vivant avec le VIH. Le but de cette étude qualitative était d'explorer et décrire des expériences parentales des couples séroconcordants qui ont un enfant tout en vivant avec le VIH au Malawi. Les données ont été collectées abuser des entrevues en profondeur avec des quatorze couples dessein échantillonnés dans les communautés matrilinéaires et patrilinéaires Chiradzulu, Chikhwawa de Juillet jeu. Décembre 2010. Les résultats de recherche montrent que, indépendamment de l'organisation de la parenté, les difficultés économiques, l'insécurité alimentaire, le rôle des attentes spécifiques de genre et des informations contradictoires des institutions de santé et les médias au sujet des sources de soutien angoisse leur rôle parental En outre, les conjoints de sexe masculin sont directement impliqués dans les activités de ménage, garde d'enfants et les décisions de l'alimentation des enfants, contestant la norme de genre stéréotypés existant. En l'absence de l'héritage des veuves, veuves des communautés patrilinéaires ne reçoivent pas le soutien attendu de la famille du mari défunt. Enfin, l'étude a montré que les couples vivant avec le VIH sont à l'aise pour trouver des solutions aux défis qu'ils rencontrent. Contrairement deux croyance existante que ce qui ont des enfants dépendent uniquement sur l'aide publique. Ces revendications sans la connaissance de contextes socioculturels locaux, peut contribuer à stigmatiser les couples vivant avec le VIH qui continuent deux ont des enfants. L'étude est également pertinent pour les personnes vivant avec le VIH qui, bien que non les parents themelves, sont confrontés à une situation où ils doivent accepter la responsabilité d'élever les enfants de leurs parents. Nous suggérons la vision à long terme de la thérapie antirétrovirale large accès au Malawi à être élargi au-delà de la fourniture d'un traitement antirétroviral deux interventions sociales et économiques Incorporer qui soutiennent la reconstruction des couples vivant avec le VIH vie sociale et économique. Les interventions doivent être conçues utilisation excessive d'une approche multi-sectorielle globale.

## Introduction

Recent data indicate that the rate of new human immunodeficiency virus (HIV) infections has decreased, for instance, in 33 countries, HIV incidence has fallen by more than 25% between 2001 and 2009 (UNAIDS 2011). Of these countries, 22 are in sub-Saharan Africa. However, there are still 22.5 million adults and children living with HIV (UNAIDS 2011).

Over the last decades, worldwide health improvements have occurred with the advent of antiretroviral therapy (ART). The result has been a dramatic reduction in HIV-related morbidity and mortality, and improvements in quality of life (Cooper, Harries, Myer, Omer & Bracken 2007; Isingo, Zaba, Martson, Ndege, Mngara, Mwita, *et al*. 2007; Kredo, Walt Van der, Siegfried & Cohen 2009; Paiva, Santos, Junior-Franca, Filipe, Ayers & Segurado 2007). HIV infection may now be considered a chronic illness (Russell, Seeley, Ezati, Wamai, Were & Bunnell 2007) because the antiretroviruses (ARVs) suppress HIV replication that results in increase in CD4 cell count, delayed clinical progression of acquired immunodeficiency syndrome (AIDS) and prolonged survival (Gallant 2000). Similarly, the number of people living with HIV (PLWH) is increasing due to decreased mortality because of ART (UNAIDS 2011). Similar situations have been noted in earlier studies in developed countries such as the USA, France, and Brazil (Attia, Egger, Müller, Zwahlen & Low 2009; Heard, Sitta, Lert & the VESPA Study Group 2007; Paiva *et al*. 2007).

ART has played an important role in decreasing perinatal HIV transmission to less than 2%, thereby reducing the women's concern regarding HIV transmission to their infants (Chasela, Hudgens, Jamieson, Kayira, Hosseinipour, Kourtis, *et al*. 2010; Kanniappan, Jeyapaul & Kalyanwala 2008). Evidence is emerging from research conducted in developing countries indicating that ART may encourage PLWH receiving treatment to reconsider their reproductive decisions such as getting married and having children (Cooper *et al*. 2007; Kredo *et al*. 2009; Matthews & Mukherjee 2009; Paiva *et al*. 2007). In some instances, ART might modify, but not eliminate, broader desires to have children (Agadjanian & Hayford 2009; Cooper *et al*. 2007; Gombachika Chimphamba, Chirwa, Sundby, Malata, Maluwa & Fjeld 2012; Hoffman, Martinson, Powers, Chilongozi, Msiska, Kachipapa, *et al*. 2008). Despite the demonstrated interest in having children amongst PLWH, the medical community has continued to be slow to support and has even discouraged PLWH in pursuing their right to bear children. Negative attitudes and biases of healthcare workers towards PLWH are reported across the world. Health workers perceptions, preferences and values have consciously or unconsciously determined the choices available to PLWH (Agadjanian & Hayford 2009; Cooper *et al*. 2007; Gruskin, Ferguson & O'Malley 2007; Kashesya Beyeza, Kaharuza, Mirembe, Neema, Ekström & Kulane 2009; Kelly, Lohan, Alderdice & Spence 2011; Newmeyer, Tecimer, Jaworsky, Chihrin, Gough, Rachlis, *et al*. 2011; Nóbrega, Oliveira, Galvão, Mota, Barbosa, Dourado, *et al*. 2007; Rutenberg, Biddlecom & Kaona 2000). As a result, couples living with HIV (CLWH) who have children are portrayed as problematic; needing support instead of addressing the challenges emanating from their decisions on their own (Enwereji & Enwereji 2010; Mahendra, Gilborn, Bharat, Mudoi, Gupta, George, *et al*. 2007; Malta, Todd, Stibich, Garcia, Pacheco & Bastos 2010).

In Malawi, ART was initiated in 2003 and since then, treatment has been available free of charge in the public sector and can be obtained at a subsidized rate from the private sector (Harries, Schouten & Libamba 2006). With these strides, deaths from AIDS have been averted by the rapid scale-up of free ARVs that has led to decline in adult mortality at the population level (Jahn, Floyd, Crampin, Mwaungulu, Mvula, Munthali, *et al*. 2008). This development raises issues not yet much explored; parenting experiences of CLWH. Literature on prevention of mother to child transmission of HIV (PMTCT) programmes has focused on the mother and the child. While literature on parenting has mostly focussed on single headed women households and elderly women as care givers (Chimwaza & Watkins 2004; Littrell, Murphy, Kumwenda & Macintyre 2012) These studies rarely provide a comprehensive understanding of how the family unit of husband and wife as CLWH experience their parenting role. The views of parents themselves have not been the focus of studies.

The objective of this study was to explore and describe parenting experiences of seroconcordant CLWH particularly those who had a child / children while living with HIV in matrilineal Chiradzulu and patrilineal Chikhwawa in Malawi. In Chikhwawa, kinship, marriage and residence are organized according to patrilineal descent, so that the transfer of, traditionally, cattle, but nowadays money (lobola) from the husband to the bride's family leading to the move of the woman from her natal household to her husband's compound after marriage, legitimizes marriage. In these compounds, men are related by patrilineal descent and the children become members of the father's homestead. This practice places a married woman in a position of dependence on her affinal kin. When the husband dies, the woman may remain in her husband's home village where she together with her children will be taken care of by the relatives of the late husband. However, the assistance provided might be inadequate as the late husband's relatives might also be providing for their families. In some situations, wife/widow inheritance is opted for where the late husband's young brother or cousin inherits the widow and cater for her and her children's needs. He could also bear children with her. However, this practice is on the decline because it enhances transmission of HIV and in some cases, surviving widows are suspected of having HIV (Cook *et al*. 1999 in Munthali 2002). If the widow is not inherited, she goes back to her home village where she may re-marry. Re-marrying for women is a form of coping (Munthali 2002).

While in Chiradzulu, the pattern is matrilineal descent and uxorilocal residence. Men leave their natal household to live in their wives' compound after marriage (Chimbiri 2007), creating compounds where children are members of the mother's homestead. The man depends on his wife's kin for land residence. In cases of divorce, children often maintain relations with their fathers throughout their life even though they may live with several of their mother's husbands. The variation seems to owe as much to personalities and circumstances as to the degree of support any father may give his children and vice versa (Peters 1997; Schwimmer 1995). In both societies, the father's contributions of work and income are essential to the proper upbringing of the child. The children are commonly raised with strong influence and support of parents as well as close relatives, friends and neighbours.

## Methodology

A qualitative approach was deemed most appropriate because there is very little existing research that has been conducted thus far. In addition, exploration of the parenting experiences of CLWH involves sensitive, emotive and personal topics that can be best captured through careful probing using qualitative in-depth interviews (IDIs).

### Study setting

Informants were recruited from ART clinics involved in the treatment and care of PLWH at two HIV and AIDS centres in Southern Malawi. The centres were Ngabu Health Centre, situated in Chikhwawa and Ndunde Health Centre, situated in Chiradzulu. These centres were chosen because they receive patients from more remote villages, away from trading centres and main roads that attract people from other districts, which was the aim of a bigger study exploring reproductive decisions made by CLWH in rural matrilineal and patrilineal communities. The informants came from catchment areas surrounding these health facilities and the average time to and from the health centres ranged from 1 to 3 h on foot. Ndunde is located 7 km from Chiradzulu district's headquarters while Ngabu is 60 km from Chikhwawa district's headquarters with an average monthly temperature of 28°C (minimum of 15.2°C, maximum of 45.6°C). Both districts face challenges ranging from food insecurity, low accessibility to safe water, low household income levels and poor communication infrastructure coupled with high prevalence of HIV and AIDS (NSO 2010). The two health facilities serve two kinship communities, patrilineal and matrilineal.

### Data collection

Informants were recruited upon receipt of permission to conduct the study following ethical approval from the research and ethics committees in Malawi, College of Medicine Research Ethics Committee and Norway, Regional Committees for Medical Research Ethics (REK). Fourteen CLWH were recruited in the study using purposive sampling. Informants who were; HIV-positive (concordant couples), had informed about each other's HIV status as a couple, in the reproductive age group of 18–49 years (NSO 2010) and had a child while living with HIV were recruited for the study. The sample size of 28 informants (14 couples) was determined based on principles of saturation and Kuzel (1992), who recommends a sample size of 12 to more data sources when trying to achieve maximum variation. Participants were sampled to saturation, and fieldwork was concluded once no new data arose from the interviews.

Data were collected from July–December 2010 and three months were spent in each study setting. This immersion and fluency in vernacular language, *Chichewa*, enabled the researcher to internalize, rather than superficially observe patterns of beliefs, fears, expectations, dominant ideas, values and behaviours of the informants (Emerson, Fretz & Shaw 1995). Practical access to the ART clinics was gained through collaboration with the ART clinic co-ordinators. In both sites, the ART clinic co-ordinators identified a contact person with whom the present study was discussed thoroughly. Considerable time was spent with the contact person to ensure that thorough comprehension was gained about the focus and the background for carrying out the study, as well as the objectives and methodological approach. The contact person oriented the researcher at the research site for a period of four days. During the orientation period, the researcher was briefed about the ART clinics' objectives, strategies in place to achieve their objectives, and activities. A pilot study to test the IDI tool was done at Chiradzulu and Chikwawa district hospitals during the researcher's orientation period to the study sites.

CLWH were approached while waiting for their monthly check-up at the two study sites. Information about the research was given to the patients of the ART clinic in two phases: as part of the general briefing that, all clients received in an open area before the check-up begins and as a private conversation with the contact person. CLWH, who indicated willingness to take part in the study, met the researcher who then asked them a few questions to determine eligibility. Simultaneous recruitment of husband and wife was not an option because couples rarely presented at the ART clinic. Therefore, in order to enhance recruitment, any married partner who indicated willingness to take part in the study, was given a two-paged letter in vernacular language, *Chichewa,* with details of the research study to give to their spouses. Researchers' interview requests were only refused where the person said he/she did not have time to participate or spouses were not available. Those eligible were accorded an appointment for an interview. Informants gave an oral consent that was recorded. Oral consent was opted for because asking them to give a written consent would have been unethical in terms of confidentiality. To further protect confidentiality, a coding system was devised to refer to each informant. The informants who consented were assured of confidentiality and informed that they were free to withdraw from the study if they so wished. No incentive was offered for participation. During the research all, the informants were provided transport reimbursements of $2 and snacks.

The researcher conducted IDIs in the vernacular language. IDIs were opted because they allow room to explore issues deeper, and are interactive in nature thereby enabling clarification of issues during the interview. In addition, they allowed further probing and modification of interview guides in the course of the study (Morse & Richards 2007). An interview guide was employed to ensure coherence in the study approach and to give the interview sessions a general direction concerning topics raised and discussed. The guide comprised a section on demographic characteristics. It also had an outline of topics with open-ended questions covering childbearing issues. The guide was carefully translated from English to Chichewa. The quality of a translation was verified by an independent translator who translated it back into the original language. Original and back translated documents were then compared for consistency by the interviewer.

Couples were interviewed independently from their spouses, but on the same day to enable free expression of feelings and views. The IDIs were carried out within the area of the ART clinic in offices or outside under trees where comfort and confidentiality were guaranteed. In order to ensure transferability, informants were asked to elaborate descriptions of their experiences and to provide a full description of the study context and setting. The interviews lasted between 50 min to 2½ h.

All recordings and data obtained are safely stored is a secure environment in locked cabinets and electronic data are password protected. They will remain safely stored for five years before being properly disposed off.

### Data analysis

**Fig. 1.**
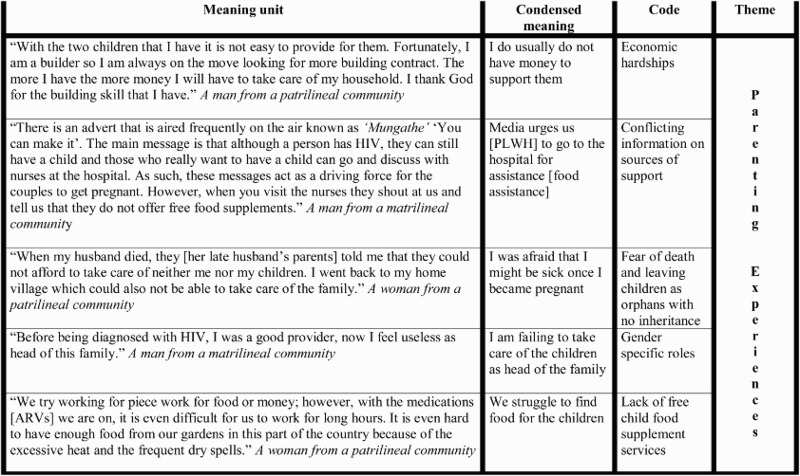
Coding process from meaning unit to theme.

The general principles and procedures for qualitative data content analysis described by Graneheim and Lundman (2004), were followed. The interviews were read through several times to obtain a sense of the whole. Then the texts about child bearing while living with HIV, were extracted which constituted the units of analysis. The texts were then divided into condensed meaning units, which were later labelled with a code. Analysis of the data resulted in 14 primary categories as indicated in Table 1. Tentative categories of the codes were discussed between three researchers who initially did the coding independently. Differences of interpretation were resolved by discussion and consensus. Four main codes; economic hardships, food insecurity, gender-specific role expectations and conflicting information from health institutions and media about sources of support reflected the most prevalent areas narrated by the couples. Once the codes were agreed upon, the underlying meaning of the different categories of the codes was formulated into a theme – parenting experiences. An example of the coding process is shown in Fig. 1. All the data from digitally – recorded IDIs that were transcribed verbatim were typed. NVivo version 9 was used to analyse and organize the data.

**Table 1.** Characteristics of the informants who had children following an HIV diagnosis.

Characteristics	Matrilineal		Patrilineal	
Age (years)	M	F	M	F
Mean	41	31	40	29
20–29	0	2	0	3
30–39	3	5	3	4
40–49	4	0	4	0
Years living with HIV				
1–5	2	2	7	7
6–10	5	5	0	0
Average number of children couples had after an HIV diagnosis	2		1	
Average number of children couples have in their households	4		4	
Couples with HIV+ children	2		0	

## Results

### Demographic and social characteristics of informants

All the 14 CLWH, seven from each community, were in a monogamous marriage relationship. They were either living in their wives or husbands' natal compounds depending on the marriage setting. Twenty-four informants had re-married, following death of a spouse (10) and divorce (14). Twenty-four of the informants were local farmers with small gardens and were without any other source of income while only four male (three and one from the matrilineal and patrilineal societies respectively) informants had formal jobs apart from owning small gardens. All the informants were Christians. Table 1 gives an overview of informants characteristics (Table 2).

**Table 2.** Primary categories.

Main categories	Other categories
• Economic hardships	• Misinformation from peers
• Food insecurity	• Disapproval from health care workers
• Conflicting information from health institutions and from media on sources of support	• Inadequate counselling from health care workers
• Gender-specific role expectations	• Stigma and discrimination
	• Embarrassment
	• Fear of transmitting HIV to the child
	• Worries (daily upkeep, death of child)
	• Sense of relief once the child is HIV-negative
	• Fear of death and leaving children as orphans with no inheritance
	• Lack of free child food supplement services

The issue of parenting experiences of CLWH reflects multiple but interrelated experiences. We limit reporting our results to four main categories; economic hardships, food insecurity, gender-specific role expectations and conflicting information from health institutions and media about sources of support. These four reflected the most prevalent areas narrated by the couples. The other remaining areas were directly related to children and the community at large, which is beyond the focus of this paper. Quotes for each category and those illustrating how informants were proactive in searching for solutions to challenges they faced have been provided.

### Economic hardships

Informants from both communities who were mostly unemployed and relied on their local gardens for produce explained that lack of finances affected their ability to provide for the needs of their child/children. They needed money for food, transport or health services. They all explained how they had to choose between going for income generating activities such as farm piecework (*ganyu* ^1^) in order to earn money for their daily household necessities and health care. In some situations health care was not a priority because of their low social-economic status. This was compounded by lack of synchrony of child services, sexual and reproductive health and ART services coupled with long travelling and waiting hours at the clinic. Some explained that when the husband, wife and child/children had different clinic appointments, ART services were prioritized over under-five clinic attendance because they felt they were lifesaving. They forgo what they labelled preventive services in order to maintain their income generating activities.

Imagine I had to wait this long just for ART services what if I had also planned to go to Family planning or escort my wife to the Antenatal clinic, I think I would need to spend 2 days here. As such I just come for the life saving drugs and off I go the other issues will be sorted out later. (A male from patrilineal community)

A woman from the matrilineal community narrated

If I miss two trips to Blantyre where I sell tomatoes, it is a big loss. I anticipate financial crisis that month. So whenever I have appointments at the ART, family planning and under-five clinic for my child on different days, I only go for the ARV clinic.

When asked what happens with her other appointments, for family planning she indicated:

I use condoms while waiting for my next injection.

While for her child's under-five clinic appointment, she narrated:

I just forgo the under-five clinic appointment since my child in now two years old and has completed all his immunizations.

The thought of feeding options for their children was a cause of great worry because most of the informants could neither afford baby formula due to economic hardship nor manage to exclusively breastfeed their babies for six months as advised by the health care workers, due to inadequate breast milk production resulting from lack of adequate food. One informant expressed with sadness her experience:

I started weaning him at six months old and I would leave him for some time at home with his sisters and come here at Makande [Main market at Ngabu in Chikwawa] to sell some local farm produce. When we run out of the tinned milk; he would take porridge or whatever was available. Unfortunately, he started getting sick frequently then became malnourished and I was also sick, I did not have enough blood in my body (anaemia). We were both admitted here for two weeks. (A woman from a patrilineal community)

For informants from the matrilineal society who had children living with HIV, the pressure of living with a child who had HIV also drained their resources in order for the child to survive, which was complicated by poverty.

Beyond the thorny issues of pure survival for the child in the midst of economic hardships, the informants were greatly concerned for their children's future. The worries, regardless of the number of children that the CLWH had and whether the children were diagnosed with HIV or not, mainly centred on the lack of care and leaving an inheritance for the child/children once they, as parents, become critically ill and eventually die. The female informants from the patrilineal community expressed their fears that following the death of their spouse or divorce, they might be unable to stay at their marital home and may be forced to go back to live with their parents and brothers. They explained that despite the expected norm for the children to remain at their late husband's home compound, the residence depended on availability of resources (human, financial and material) of the parents and brothers in-law. Therefore it was not surprising for some of these women in case of divorce or death of their spouse to anticipate to be told to either leave their children behind or take them along, thus putting them in a difficult economic situation as narrated below by a women in her second marriage.

When my first husband died, they [her late husband's parents] told me that they could not afford to take care of neither me nor my children. I went back to my home village, which was also poor. I struggled to take care of my children since most of our resources had been used up during my husbands’ illness.

Unlike the male informants from the matrilineal community who indicated that following the death of their spouses, it was very unlikely to be given custodian of the children. They had to move back to their homes leaving the children behind regardless of social-economic status of their wives kin.

For most of these informants, planning meant consideration of guardianship, another complex and difficult issue they expressed. The informants often doubted that the extended family, which has been considered a safety net in their communities, might provide adequate financial support to the children. The primary reason given for the lack of support from the extended family was poverty.

As a means of overcoming the economic challenges, some informants explained that they would venture in small-scale income-generating activities. They cited piecework in gardens (*ganyu*), often in exchange for money or small portions of maize flour, selling some of their farm produce, and to the male informants who had technical jobs, they indicated how they intensified their search for extra contracts, which would bring more income for them.

With the two children that I have it is not easy to provide for them. Fortunately, I am a builder so I am always on the move looking for more building contract. The more I have the more money I will have to take care of my household. I thank God for the building skill that I have. (A man from a patrilineal community)

A woman from a matrilineal community explains how their family managed to overcome their economic hardships;

My husband was weak most of the times to work in our garden and I would always rely on my mother and sisters for support. Things went bad when the assistance would be accompanied with insults; ‘do you bear children for us to be assisting you’ until one day I told myself that enough is enough. We discussed with my husband and I started buying tomatoes from local farmers around our village. I would then sell them in town (Blantyre). That's how we have survived the economic hardship all these years.

### Gender-specific role expectations

Fuelling the experiences of economic hardships were the issues of expected social cultural gender-specific roles. The narratives revealed a noticeable difference in the role men and women assume in the family unit as narrated by a woman from the matrilineal community;

You know, the problem in our village is we [women] depend a lot on our husbands. We always wait for the husband to go for piecework [ganyu] in someone's garden; get paid, buy some food and bring it home. If you have disagreed with him that day and you do not have food in the house, it means you will not eat until when you reconcile.

Male informants narrated their lack of being able to fulfil the provider role, as expected in their community mainly due to; either lack of money or food and physical strength to work in the gardens. As head of the household living with HIV had made them, have difficulties to maintain an income to adequately provide for their family. Male informants specifically stated the idea that men are responsible for the upkeep of their families on numerous occasions, for example;

I am failing to take care of the children as head of the family. (A man from a patrilineal community)

It is my responsibility to provide for my family. (A man from a patrilineal community)

Before being diagnosed with HIV, I was a good provider, now I feel useless as head of this family. (A man from a matrilineal community)

Female informants from the matrilineal community, unlike their counterparts from the patrilineal community, narrated how their spouses had changed since they were diagnosed with HIV. The spouses were able to fulfil some of the gender-specific roles within the family. Some of the roles that they mentioned were; taking the child to the under-five clinic, ART clinic, cooking for the family and bathing the children when their mother was away or sick, and involvement in child feeding decisions. The women indicated that they were pleased about these changes in their family life.

### Food insecurity

The third major theme that emerged from the interview sessions was food insecurity. Informants indicated that they were food insecure and expressed that they would benefit from food supplements for the child/children. Although all the informants shared common concerns, their experiences were diverse depending on geographical setting. For instance, informants from Chikhwawa narrated how difficult it was for them to have food and could hardly afford one full meal a day since the district is prone to droughts.

We try working for piece work [ganyu] for food or money; however, with the medications [ARVs] we are on, it is even difficult for us to work for long hours. It is even hard to have enough food from our gardens in this part of the country because of the excessive heat and the frequent dry spells. (A woman from a patrilineal community)

Unlike their counterparts in Chiradzulu who though also poor, could afford two full meals a day.

In fact we do not buy most of the food because we get it from our garden. Vegetables we have, pigeon peas we have, eggs we have, a hen can lay 16 eggs so we decide to have some of them and leave the rest for hatching. At least we have the food. When we do not have, we go for farm piece work [ganyu] for money. (A woman from a matrilineal community)

Some of the informants sought help too by asking about resources and making contacts with relevant services. They indicated that seeking help needed determination and most of the times not always a comfortable experience as it was often misinterpreted as begging. One couple from a matrilineal community explained how they managed to get food supplements for their child from a private non-profit-making organization.

During one of our support group meetings (PLWH) at PIM [Providence Industrial Mission], I was surprised to see my friends preparing tinned milk for their children. Upon inquiry, they told me that they get it free at Nguludi from a European woman who has a charitable organization for the underprivileged. She told me the direction to the place; it was far a day's travel by bicycle. I told my husband. A week later, we went to Nguludi. When we arrived, we were introduced to the woman and we were surprised that she was able to speak ‘Chichewa’ local language. We told her that we were seeking for food supplements for our 5 months old child because we were HIV positive and poor. She took our particulars and asked for a confirmation that we were living with HIV. We showed her our health passport books. We were registered the same day along with several others and we were given the food supplements; lactogen 1, maize flour and some baby clothes. Since that time we would go for the free food supplements until when our son was 3 years. (A woman from a matrilineal community)

### Conflicting information on sources of support

Information from health institutions and from the media about sources of support was a challenging experience for the couples. They indicated that there is a lot of information on the radio about PLWH and sources of support. One such programme was the ‘Mungathe’ ‘It is possible’. This programme advocates that it is possible for PLWH to have HIV-negative children provided they follow stipulated guidelines. The informants further indicated that the media urges PLWH to go to the hospital for free advice and assistance. To them the issue of assistance translates to provision of food supplements to children, counselling and psychological support among many others. However, those who managed to express their needs to the hospital discovered that their respective health facilities do not offer food supplements to children born to PLWH. Neither do they have services in their local communities, which offer such services free. The majority also indicated receiving advice from the healthcare workers against becoming pregnant. Informants also mentioned judgemental tones from the healthcare workers because of not following the advice on pregnancy prevention. A man from a matrilineal community narrates:

There is an advert that is aired frequently on the air known as ‘Mungathe’ ‘You can make it’. The main message is that although a person has HIV, they can still have a child and those who really want further information and assistance can go and discuss with nurses at the hospital. However, when you visit the nurses they shout at us, accuse us of having children and tell us that they do not offer free food supplements.

## Discussion

This study explored parenting experiences of CLWH who had a child or children while living with HIV in rural matrilineal Chiradzulu and patrilineal Chikhwawa districts in Southern Malawi. The challenges that these couples face as they take care for their children can be categorized as; economic hardships, food insecurity, gender-specific role expectations and conflicting information from health institutions and media about sources of support. Nevertheless, their experiences were diverse depending on gender, geographical setting and kinship organization.

### Economic hardships and food insecurity

The findings show that poverty and food insecurity remain an overriding challenge for most couples because they carry a double burden on one hand and food insecurity on the other in resource-constrained settings such as sub-Saharan Africa (Himmelgreen, Daza-Romero, Turkon, Watson, Uma-Okello & Sellen 2009; Hosegood, Preston-White, Busza, Moiste & Timaeus 2007; Kadiyala & Gillespie 2004; Kindra, Coutsoudis & Esposito 2011).

Only four male informants had formal jobs. Most of the couples were local farmers with small gardens and were without a reliable source of their own income and they relied on *ganyu* whose remuneration is low. Although many people in Malawi engage in *ganyu*, it is to some extent shameful as it is an admission of poverty and sign that the household does not have any food (Whiteside 2000). Usually *ganyu* robs the couples' time to take care of their children and tend to their gardens (Mkandawire & Ferguson 1990 in Hosegood *et al*. 2007). It is, therefore, not surprising that despite *ganyu,* male informants expressed concerns about their failure to provide for their families. In both communities, it is the husbands' duty to provide for the family; the ability to provide for the family is one of the main pillars upon which the social representation of masculinity is constructed (Bila & Egrot 2009). Failure to provide for the family represents a shame in the cultural context of these two communities.

Women from patrilineal community were not welcome to remain in their deceased husband's compound because either most of the economically active adults who could offer support have already died or their in-laws were also poor due to depletion of their productive and financial assets from earlier livelihood to cover expenditures incurred due to long-term illness (Russell 2004). Suggesting that although community systems were expected to offer support, community dynamics seem to have changed with the economic times and HIV epidemic (Wachira, Middlestad, Vreeman & Braitstein 2012).

Linked with the issue of food insecurity was the experience of breastfeeding and replacement feeding. The experience was of substantial concern particularly amongst couples from Chikhwawa. Apart from its worst health indicators, climatic and geographical features of Chikhwawa contribute to food insecurity. Almost every year the area is faced with dry spells and floods from Shire river and its tributaries leading to a reduction in the overall agriculture production (Chunga, Jabu, Taulo & Grimason 2004; Malawi Government 2010).

We are aware that if these challenges are not addressed may lead to high risks of child mortality, HIV-related morbidity, increased hospitalizations and decreased utilization of outpatient care services (Weiser, Tsai, Gupta, Frongillo, Kawuma, Senkungu, *et al*. 2012). Therefore, we suggest a longer-term vision for ART wide access in Malawi to be broadened to go beyond provision of ARVs (Russell *et al*. 2007) to incorporate social and economic interventions that support the rebuilding of CLWH's social and economic lives. We support the proponents of Kalichman, Pellowski, Kalichman, Cherry, Detorio, Caliendo, *et al*. (2011) and Weiser *et al*. (2012), that social and economic interventions must be an integral component of the HIV programmes serving impoverished populations in Malawi.

### Conflicting information on sources of support

Conflicting messages on sources of support has been expressed as a challenge consistent with Gombachika Chimphamba *et al*. (2012). In order to address the situation, we suggest that before any information pertaining to sexual and reproductive health, HIV and AIDS is produced, it must be thoroughly scrutinized and compared with existing information. Such a responsibility should be co-ordinated by the main Malawi government health providers, Ministry of Health.

### Gender-specific role expectations

The findings suggest that male spouses are directly involvement in household activities, childcare and child feeding decisions, contrary to stereotyped gender norms reported in Burkina Faso (Bila & Egrot 2009), Kenya (Wachira *et al*. 2012), Zimbabwe (Skovdal, Campbell, Madanhire, Mupambireyi, Nyamukapa & Gregson 2011), Malawi (Chimwaza & Watkins 2004; Chipeta 2009). Men and women do not share the same spaces; male–female boundaries are strictly observed, and each sex performs the work incumbent upon it. Any transgression of the gender boundary is therefore, seen as inappropriate or incongruous. However, the findings are consistent with what Kebaabetswe (2007), Moth, Ayayo and Kaseje (2005), and Tijou, Querre, Brou, Leroy, Desclaux, Desgrées-du-Loû, *et al*. (2009) reported that male involvement is associated with increased ease of uptake of exclusive breastfeeding and other PMTCT activities.

Notably, the findings suggest that CLWH attempt to find solutions for the challenges they encounter during parenting. Contrary to what some literature cites about PLWH who have children, few resources at their disposal, as highly dependent on state aid (Åsander, Belfrage, Pehrson, Lindstein & Björkman 2004; Oluwagbemiga 2007; Zhang, Zhang, Aleong, Baker & Fuller-Thomson 2012), Such claims, we argue, contribute to stigmatize CLWH who continue to have children due to the wider access to reproductive options. The stigma emanating from such claims might lead to unwillingness to seek help and access health services (Holzener & Uys 2004) and interfere with HIV and AIDS preventive services currently instituted in Malawi.

Finally, the analysis of the four themes governing parenting experiences by CLWH in this study can be framed in the Sallis and Owen (2002) socio-ecological model (SEM) that emphasizes the dynamic interaction between an individual and the environment. The model recognizes that: where individuals are responsible for instituting and maintaining life style changes necessary to reduce risk and improve health, individual behaviour is influenced by factors at different levels (Elder, Lytle, Sallis, Young, Steckler, Simons-Morton, *et al*. 2007). In this paper, we have shown that informants' challenges; economic hardships, food insecurity, gender-specific role expectations and conflicting information from health institutions and media about sources of support, could fall across SEM levels (individual, interpersonal, organization, community and societal). The complex interrelationship between these suggests a holistic multi-sector approach to address the challenges.

This study focuses on CLWH, consequently, enabling us to explore how the experiences of a husband and a wife are unified to produce one-outcome socio-cultural contexts. However, our findings and recommendations should be read against the fact that the study was conducted in rural settings, used purposive sampling procedure, which could have resulted to selection bias. Couples who self-selected to enrol in the study may differ from other couples in a number of ways. They may be more disclosive and connected to HIV-related services than other CLWH. Larger scale longitudinal mixed method studies that follow matrilineal and patrilineal CLWH who have just delivered their children until they are 24 months are needed.

## Conclusion

The findings provide new insight into the parenting experiences of CLWH, a timely issue, given the growing interest of parenting by PLWH due to wide access to ARVs. In addition, understanding the parenting experiences of CLWH, and gaining their insights into their needs and concerns are essential steps to providing supportive care. The study is also relevant to PLWH who, although not parents themselves, may accept responsibility of raising children from their kin. Economic hardships, food insecurity, gender-specific role expectations and conflicting information from health institutions and media about sources of support were underpinning their parenting roles. The study has shown that male spouses are directly involved in household activities, childcare and child feeding decisions, challenging the existing stereotyped gender norms. In addition, the study has shown that CLWH are able to find solutions for the challenges they encountered. Contrary to existing belief that CLWH who have children depend solely on public aid. Such claims without proper knowledge of local social cultural contexts, may contribute to stigmatizing CLWH who continue to have children. Our analysis reveal that factors associated with parenting in CLWH are nested within the intrapersonal, interpersonal, organizational, community and societal levels of the SEM. Therefore, we suggest first, that the longer-term vision for ART wide access in Malawi incorporate social, cultural and economic interventions that support the rebuilding of CLWH social and economic lives. Second, use of a holistic multi-sector approach in designing intervention programmes. However, the findings are limited to concordant couples CLWH from the two study settings.

## **Note**

1. Informal work undertaken to earn food and/or cash for survival, ranging from work on agricultural estates to short term exchanges of labour for food or wages (Whiteside 2000).
